# Worker Characteristics and Measures Associated With Patient and Visitor Violence in the COVID-19 Pandemic: A Multilevel Regression Analysis From China

**DOI:** 10.3389/fpubh.2022.877843

**Published:** 2022-06-02

**Authors:** Ya-qian Guo, Ju Huang, Na-na Xu, Xiao-jing Ma

**Affiliations:** ^1^Institute of Medical Information, Chinese Academy of Medical Sciences and Peking Union Medical College, Beijing, China; ^2^The Second Medical Center & National Clinical Research Center for Geriatric Diseases, Chinese PLA General Hospital, Beijing, China

**Keywords:** COVID-19, health workers (HWs), patient and visitor violence, workplace violence, multilevel logistic regression

## Abstract

**Objective:**

To analyze the patient and visitor workplace violence (PVV) toward health workers (HWs) and identify correlations between worker characteristics, measures against violence and exposure to PVV in COVID-19 pandemic.

**Methods:**

A cross-sectional survey utilizing the international questionnaires in six public tertiary hospitals from Beijing in 2020 was conducted, and valid data from 754 respondents were collected. Multilevel logistic regression models were used to determine the association between independents and exposure to PVV.

**Results:**

During COVID-19 pandemic and regular epidemic prevention and control, doctors were 5.3 times (95% CI = 1.59~17.90) more likely to suffer from physical PVV than nurses. HWs most frequently work with infants were 7.2 times (95% CI = 2.24~23.19) more likely to suffer from psychological PVV. More than four-fifth of HWs reported that their workplace had implemented security measures in 2020, and the cross-level interactions between the security measures and *profession* variable indicates that doctors in the workplace without security measures were 11.3 times (95% CI = 1.09~116.39) more likely to suffer from physical PVV compared to nurses in the workplace with security measures.

**Conclusion:**

Doctors have higher risk of physical PVV in COVID-19 containment, and the security measures are very important and effective to fight against the physical PVV. Comprehensive measures should be implemented to mitigate hazards and protect the health, safety, and well-being of health workers.

## Instruction

The COVID-19 pandemic has very clearly revealed the huge challenges and risks facing health workers (HWs) globally. Violence and harassment against health workers have been increasing during the COVID-19 pandemic (1–3). Experience shows that stress and fatigue, long patient waiting time, crowding, COVID-19-specific prevention, and control measures (such as placing individuals in quarantine or isolation facilities), contact tracing etc., are most widespread risk factors for workplace violence in the health sector and can lead to acts of violence against healthcare professionals and others who directly care for patients and their visitors (3, 4). Violence, harassment, discrimination, and stigma from patients and their visitors against health workers should be prevented and eliminated as much as possible.

In China, proactive policies and measures were issued and implemented for HWs by the ministries and commissions of Chinese government, until the mid of March 2020, not only including the infection prevention and control (IPC) measures, but also measures of improving working condition and caring for physical and psychological health for HWs (5–7). In June 2020, COVID-19 broke out in Beijing, and 337 infected cases have been reported in 25 days. After that, all hospitals in Beijing have tightened IPC measures, and implemented proactive occupational health measures and preventive strategies against occupational hazards in hospitals. For example, a large number of public hospitals had set up security check system which was practically non-existent prior to the outbreak of COVID-19 (8). These policies and actions generally provide HWs qualified personal protective equipment, good work organization, and prevention strategy of violence and discrimination.

For many years, the prevalence and risk factors associated with PVV against HWs have been studied worldwide, previous studies discovered individual characteristics of perpetrator and victim (9–11), the HWs-patient and visitor interactions (12), the characteristics of the work environment (10, 13), and the official organizational hospital policies (14) seem important in the occurrence of PVV (15). Recently, several studies have investigated the occurrence of workplace violence (WPV) against HWs during COVID-19 pandemic in China, estimating that the percentage of experienced WPV against HWs was from 17.9 to 20.4% during the COVID-19 outbreak (<1 year), and risk factors have been identified for individual characteristics of HWs (16). What's more, public health studies indicate that individual health behavior and outcomes are jointly determined by individual and environmental factors (17). Risk factors of PVV may be associated with individual level factors, such as age, gender, profession, experience, as well as the hospital setting in which the HWs are imbedded, the contextual factors, such as the geographic location, institutional scale and type, and existing measures. However, to our knowledge, research on the causes and factors related to PVV during the epidemic is limited and fragmented, and there is little research which explores measures to deal with PVV and the effect of cross-level interactions between individual factors and the measures on PVV (18), by the questionnaire—*Workplace Violence in the Health Sector Country Case Studies Research Instrument-Survey Questionnaire* (hereafter referred to as “the international questionnaires”), which were jointly developed by ILO, the International Council of Nurses (ICN), the WHO and the Public Services International (PSI) (19). In addition, the literature reveals a lack of studies describing a coherent, non-fragmented analysis of the situations where PVV occurs and which is based on the experiences of different professions working in a variety of units from multiple hospitals.

The world is in the grip of the COVID-19 pandemic and the new mutation spreads more readily than the original, thus the health states of HWs should be valued, and violence, harassment, discrimination, and stigma from patients and their visitors against HWs should be prevented and eliminated as much as possible. In this study, 12-month prevalence of PVV in Beijing from 1 January to 31 December, including the period of COVID-19 pandemic, were described, correlations between worker characteristics, measures, the cross-level interactions, and exposed to PVV were examined. The findings may provide evidence on occupational health and safety measures for HWs and occupational health services in the context of the COVID-19 pandemic.

## Methods

A cross-sectional survey utilizing the international questionnaires was conducted in January 2021 and included HWs from six public tertiary hospitals in Beijing.

### Sample

The sampling strategy was divided into two steps. Firstly, two hospitals were selected in east, west, and north of Beijing, respectively. We purposefully sampled general and specialized hospitals. Then we used convenience sampling to recruit participants. Under the coordination of the managerial department of each hospital, investigators first obtained permission from the departments where most of the HWs were willing to participate in the survey. At least two departments were investigated in each hospital, and the participating departments almost covered all major types of wards. All the HWs on duty were invited to fill in the questionnaire during the survey time from 8 a.m. to 5 p.m. in 1 day for each department. The inclusion criteria for HWs were those working in direct contact with patients/visitors and full-time employees of these public hospitals with qualification certificates. The sample included the following professions: physicians, nurses and midwives, pharmacists, physical therapists, occupational therapists, and dieticians, technical staff (e.g., laboratory/sterilization workers), and administrative staff.

The participating hospitals are all large, tertiary public hospitals. Five of them are general hospitals, and four general hospitals and one specialized hospital are university hospitals. The specialized hospital is a child hospital. The description of characteristics (number of beds, total workers) of each hospital is presented in Table 1. Eight hundred and fifty-nine HWs from the selected department of these hospitals met the inclusion criteria, and 760 of them participated in the survey voluntarily, of whom 754 returned valid questionnaires (total valid response rate 87.8%) (Table 1). 84.5 and 15.5% of the respondents were from general and specialized hospitals, respectively. The sample of HWs is typical for HWs in the health sectors of Beijing according to gender (87.4% vs. 74.3% female), age (about 60% vs. 50% over 35 years old), professional experience (about 65% over 6 years vs. 79.5% over 5 years), and department (20).

**Table 1:** Description of hospital characteristics and the hospital-level prevalence of PVV.

	**Beds**	**Total no. of workers**	**Valid respondents (%)**	**Included HWs** **(%)**	**No. of HWs experience PVV (%)**		
					**Overall**	**Psychological**	**Physical**
General hospital	–	–	722 (84.1)	637 (84.5)	172 (27.0)	170 (26.7)	17 (2.7)
Hospital 1	1,600	3,389	138 (16.1)	121 (16.0)	41 (33.9)	41 (33.9)	7 (5.8)
Hospital 2	1,500	2,954	129 (15.0)	114 (15.1)	36 (31.6)	35 (30.7)	2 (1.8)
Hospital 3	1,500	2,498	186 (21.7)	166 (22.0)	48 (28.9)	48 (28.9)	7 (4.2)
Hospital 4	1,500	4,224	123 (14.3)	110 (14.6)	30 (27.3)	29 (26.4)	1 (0.9)
Hospital 5	800	1,653	146 (17.0)	126 (16.7)	17 (13.5)	17 (13.5)	0 (0.0)
Specialized hospital	–	–	137 (15.9)	117 (15.5)	48 (41.0)	47 (40.2)	14 (12.0)
Hospital 6	400	2,662	137 (15.9)	117 (15.5)	48 (41.0)	47 (40.2)	14 (12.0)
Total	–	–	859 (100.0)	754 (100.0)	220 (29.2)	217 (28.8)	31 (4.1)

*PVV, patient and visitor violence*.

The cross-sectional survey was carried out in January 2021 and took 1 month to complete. The 12-month workplace violence before the investigation time points (from 1 January 2020 to 31 December 2020, covering the period of COVID-19 outbreak in Beijing) was investigated. HWs participating in the survey received written information about the study's aim, background, and voluntary nature of participation.

### Instrument

Workplace Violence in the Health Sector Country Case Studies: Survey Questionnaire, Chinese Version-Revised (the international questionnaires-C-R) (21) was employed for data collection. The international questionnaires-C-R is based on the English version developed by ILO, ICN, WHO, and PSI, and was translated and tested for public hospitals by using in the Chinese language (22). The questionnaire includes the following four parts: personal and workplace data (individual characteristics), physical workplace violence, psychological workplace violence (verbal abuse, bullying/mobbing, sexual harassment, and racial harassment), and health sector employer information. The item of measures to deal with workplace violence existing in the workplace belongs to the part of health sector employer. The comprehensibility and validity of the international questionnaires-C-R was tested by some studies involving different health professions from different kinds of hospitals in China (21–25). The Cronbach's coefficient is 0.828 in this study, and our analysis suggested that the international questionnaires-C-R was comprehensible, comprehensive and meaningful for actual practice in China's public hospitals.

### Variable Description

The multi-level data in this study includes two level variables: demographic variables (level-1) and contextual variables of the measures (level-2). Contextual variables are generated from original individual level data.

The analysis centers on two level-1 outcomes variables (dichotomous measures): Psychological PVV and Physical PVV. The covariates are all dummy variables: age, gender, profession, experience, department, and patients/clients most frequently work with variables. The level-2 contextual variable is *measure (n)*, which presents the 13 existing measures against violence and listed in **Table 4**. *Measure (n)* is a dummy variable (1—The measure (*n*) existed in HWs' workplace; 0—The measure (*n*) didn't exist in HWs' workplace), and *n* = 1, 2,…, 13.

### Statistical Analysis

Because of the hierarchical structure of the data and the discrete outcome, multilevel logistic regression analysis, a type of the Generalized Linear Mixed Model (26), was used to assess the association between the independents and PVV. Cross-level interactions in multilevel modeling enable us to assess the degree to which relationships between individual explanatory and outcome variables are moderated by group level variables. In addition, the assumption of observation independence is not required in multilevel modeling because multilevel models are designed to measure and thus account for ICC (Intra-class Correlation Coefficient) in hierarchically structured data (26). Therefore, this study established a Two-Level Logistic Regression Model, with the individual (including outcome measure and individual variables) as level-1 and the measures as level-2.

The results of empty model for psychological PVV and physical PVV showed that the variance of each intercept was statistically significant (ICC = 0.16, chibar2 (01) = 11.0, *P* = 0.0005) (ICC = 0.26, chibar2 (01) = 13.2, *P* = 0.0001). And the ICC showed a moderately large between-group heterogeneity or within-group heterogeneity. Thus, the multilevel modeling approach should be applied to this data. The basic two-level logistic model is as follows:


$$\begin{array}{l} \ln\left(\frac{P_{ij}}{1-P_{ij}}\right)=\beta_{0j}+\beta_{1j}Profession+\beta_{2}Gender_{ij}+\beta_{3}Age_{ij}\\ \;+\beta_{4}Experience_{ij}+\beta_{5}Ward_{ij}+\beta_{6}Patients_{ij}\left(I\right)\\ \;\beta_{0j}=\gamma_{00}+\gamma_{01}Measures{\left(n\right)}_{j}+\mu_{0j}\left(II\right)\\ \;\beta_{1j}=\gamma_{10}+\gamma_{11}Measures{\left(n\right)}_{j}+\mu_{1j}\left(III\right)\\ \ln\left(\frac{P_{ij}}{1-P_{ij}}\right)=\gamma_{00}+\gamma_{01}Measure{\left(n\right)}_{j}+\gamma_{10}Profession_{ij}\\ \;+\gamma_{11}Measures{\left(n\right)}_{j}*Profession_{ij}\\ \;+\beta_{2}Gender_{ij}+\beta_{3}Age_{ij}+\beta_{4}Experience_{ij}\\ \;+\beta_{5}Ward_{ij}+\beta_{6}Patients_{ij}\\ \;+\left(\mu_{0j}+\mu_{1j}*Profession_{ij}\right)\left(IV\right) \end{array}$$

Equation (I) is the level-1 equation. Equation (II) and (III) illustrate the case of two level-2 equations. Where *p*_*ij*_ represents the probability of PVV occurring. The *i* represents the *i*^*th*^ individual (level-1 unit), and *j* presents the *j*^*th*^ hospital (level-2 unit); *i*=*1, 2,…, N* (*N* is the total sample size), and *j*=*1,2,…,J* (*J* is the number of hospitals). Equation (*IV*) is a combined model, and (μ_0j_$+\mu_{ij}^{*}$Profession_*ij*_) is the composite error term. A new variable $Measure_{j}^{*}$Profession_*ij*_ was created, which denotes the cross-level interaction between the contextual variable *Measure* and the level-1 variable *Profession*.

Descriptive statistics were used to describe demographic and frequency of psychological and physical PVV, and existing measures against violence (MAV) was evaluated. Associations between categorical variables were tested with chi-square tests. All data analyses were conducted using Stata 16.0. Statistical significance was defined as *p* < 0.05.

## Results

### Prevalence and Distribution of PVV

Of the 754 respondents who completed valid questionnaires in the survey, 87.4% were women and 56.6% were between 35 and 45 years old (the mean age of total participants was 32.6 ± 6.8 years), 82.6% were nurses and 15.1% were doctors, and 32.8% had between 6 and 10 years of experience at the health sector (Table 2).

**Table 2:** Characteristics and frequency distributions for PVV among 754 HWs.

		**No. of HWs** **(%)**	**Overall PVV**		**Psychological PVV**		**Physical PVV**	
			**No. of HWs (%)**	***X^**2**^* (*p*)**	**No. of HWs** **(%)**	***X^**2**^* (*p*)**	**No. of HWs (%)**	***X^**2**^* (*p*)**
Total		754 (100.0)	220 (29.2)		217 (28.8)		31 (4.1)	
Gender	Female	659 (87.4)	202 (30.7)	5.51	200 (30.3)	6.28	20 (3.0)	15.38
	Male	95 (12.6)	18 (18.9)	(0.019)	17 (17.9)	(0.012)	11 (11.6)	(0.000)
Age	Under 35	297 (39.4)	81 (27.3)	1.64	78 (26.3)	2.30	11 (3.7)	0.63
	35~45	427 (56.6)	132 (30.9)	(0.44)	132 (30.9)	(0.317)	18 (4.2)	(0.728)
	46 and older	30 (4.0)	7 (23.3)		7 (23.3)		2 (6.7)	
Profession	Nurse	623 (82.6)	199 (31.9)	13.43	197 (31.6)	14.24	18 (2.9)	18.47
	Doctor	114 (15.1)	19 (16.7)	(0.001)	18 (15.8)	(0.001)	13 (11.4)	(0.000)
	Other HW	17 (2.3)	2 (11.8)		2 (11.8)		0 (0.0)	
Experience in health sector	<1 year	33 (4.4)	4 (12.1)	7.83	3 (9.1)	9.76	2 (6.1)	6.78
	1–5 year	227 (30.1)	66 (29.1)	(0.166)	64 (28.2)	(0.082)	14 (6.2)	(0.238)
	6–10 year	247 (32.8)	72 (29.1)		72 (29.1)		10 (4.0)	
	11–15 year	142 (18.8)	49 (34.5)		49 (34.5)		3 (2.1)	
	16–20 year	60 (8.0)	19 (31.7)		19 (31.7)		0 (0.0)	
	Over 20 years	45 (6.0)	10 (22.2)		10 (22.2)		2 (4.4)	
Department	OED	184 (24.4)	56 (30.4)	22.88	54 (29.3)	23.24	9 (4.9)	8.13
	General medicine	270 (35.8)	69 (25.6)	(0.000)	68 (25.2)	(0.000)	6 (2.2)	(0.149)
	General surgery	85 (11.3)	31 (36.5)		31 (36.5)		5 (5.9)	
	Intensive care	54 (7.2)	12 (22.2)		12 (22.2)		2 (3.7)	
	Specialized unit	124 (16.4)	50 (40.3)		50 (40.3)		9 (7.3)	
	Support services	37 (4.9)	2 (5.4)		2 (5.4)		0 (0.0)	
Work in nights	Yes	352 (46.7)	108 (30.7)	0.72	107 (30.4)	0.84	18 (5.1)	1.68
	No	402 (53.3)	112 (27.9)	(0.395)	110 (27.4)	(0.359)	13 (3.2)	(0.195)
Patients/clients most frequently work with	Newborns	31 (4.1)	8 (25.8)	0.18	8 (25.8)	0.14	3 (9.7)	2.54
				(0.673)		(0.709)		(0.111)
	Infants	26 (3.4)	19 (73.1)	25.11	19 (73.1)	25.78	6 (23.1)	24.57
				(0.000)		(0.000)		(0.000)
	Children	88 (11.7)	54 (61.4)	49.95	54 (61.4)	51.61	8 (9.1)	6.27
				(0.000)		(0.000)		(0.012)
	Adolescents	91 (12.1)	47 (51.6)	25.29	45 (49.5)	21.57	7 (7.7)	3.37
				(0.000)		(0.000)		(0.067)
	Adults	466 (61.8)	153 (32.8)	7.89	151 (32.4)	7.82	18 (3.9)	0.19
				(0.005)		(0.005)		(0.662)
	Elderly	393 (52.1)	129 (32.8)	5.28	129 (32.8)	6.55	13 (3.3)	1.34
				(0.022)		(0.01)		(0.246)

*PVV, patient and visitor violence*.

*OED, Outpatient and emergency department*.

*Doctor: physicians, pharmacists, physical therapists, occupational therapists, and dietitians*.

*Nurse: nurse and midwife*.

*Other HWs: technical staff (e.g., laboratory/sterilization workers) and administrative staff*.

*Specialized unit: psychiatric, pediatrics, orthopedics, radiology*.

In 2020, a total of 220 (29.2%) HWs experienced the PVV. 217 (28.8%) and 31 (4.1%) respondents have witnessed incidents of psychological and physical PVV in their workplace, respectively. 10.2% of respondents reported the psychological PVV occurred 2–4 times in the 12 months of 2020. Table 1 presents the hospital aggregates from the respondent data described in the text. The prevalence of psychological and physical PVV for specialized hospitals (40.2 and 12.0%) were both higher than that of general hospitals (26.7 and 2.7%) (all *p* < 0.01).

Across occupations, nurses had the highest exposure to PVV, followed by doctors and other HWs (technical and administrative staff) (31.9% vs. 16.7% vs. 11.8%) (χ^2^= 13.4, *p* = 0.001). The doctor had the highest exposure to physical PVV (11.4%), while nurses had the highest exposure to psychological PVV (31.6%). When comparing the occurrence of PVV in different departments, the prevalence is highest in specialized unit (e.g., psychiatric, pediatrics, orthopedics, and radiology units), followed by general surgery and outpatient and emergency department (40.3% vs. 36.5% vs. 30.4%) (χ^2^ = 22.9, *p* < 0.001). In addition, the prevalence of PVV for HWs who frequently work with infants (73.1%) (χ^2^ = 25.1, *p* < 0.001), children (61.4%) (χ^2^ = 50.0, *p* < 0.001), and adolescents (51.6%) (χ^2^ = 25.3, *p* < 0.001) were higher than they frequently work with other patients/clients (Table 2). No significant between-group difference was found for age, work experiences, and night shift (all *p* > 0.05).

### Multilevel Logistic Regression of Occupational Characteristics

Model 1.1 and 1.2 in Table 3 shows the results of multilevel logistic regression to determine the association between demographic indicator variables and PVV, by a combined model of Equation (I) and empty model of level-2^1^. It indicates that after controlling for HWs characteristics, professions, department, and patients/clients most frequently work with are related to the occurrence of PVV. Nurses had a greater risk of psychological PVV than doctors who were 0.5 times (95% CI = 0.23~0.99) less likely to suffer from psychological PVV than nurses, while doctors were 5.3 times (95% CI = 1.59~17.90) more likely to suffer from physical violence than nurses. HWs in General surgery (OR = 2.3, 95% CI = 1.22~4.40) and intensive care (OR = 22.9, 95% CI = 2.90~181.23) had a greater risk of psychological PVV and physical PVV than in general medicine, respectively. Those who most frequently work with infants were 7.2 times (95% CI = 2.24~23.19) more likely to suffer from psychological PVV than HWs most frequently work with other patients. No important association was found between having experienced PVV and gender, age, and length of experience in the health sector (Table 3).

**Table 3:** Results of multilevel logistic regression models: worker characteristics, measures and cross-level interactions (*n* = 754).

**Variable**		**Model 1.1** **psychological PVV**	**Model 1.2** **physical PVV**	**Model 2.1** **psychological PVV**	**Model 2.2** **physical PVV**
		**OR (95% CI)**	**OR (95% CI)**	**OR (95% CI)**	**OR (95% CI)**
**Level-1**					
Gender	Male	0.86 (0.42~1.77)	3.07 (0.94~9.99)	0.97 (0.49~1.92)	3.30 (0.97~11.26)
	Female	1.0	1.0	1.0	1.0
Age	35~45	1.21 (0.74~1.98)	1.68 (0.56~5.04)	1.17 (0.73~1.87)	2.03 (0.69~6.01)
	46~	1.30 (0.38~4.51)	4.37 (0.41~46.15)	1.27 (0.4~4.06)	4.34 (0.40~47.62)
	Under 35	1.0	1.0	1.0	1.0
Profession	Doctor	0.48 (0.23~0.99)^a^	5.34 (1.59~17.90)^b^	0.31 (0.03~2.86)	1.49 (0.35~6.32)
	Other HWs	0.63 (0.12~3.37)	–	15.64 (0.87~282.37)	–
	Nurse	1.0	1.0	1.0	1.0
Experience in health sector	under 1 year	0.38 (0.07~1.92)	2.28 (0.13~39.06)	0.47 (0.1~2.3)	1.74 (0.10~30.94)
	1–5 year	1.92 (0.67~5.49)	2.62 (0.29~23.64)	1.73 (0.62~4.83)	2.27 (0.24~21.61)
	6–10 year	1.78 (0.68~4.61)	1.11 (0.15~8.49)	1.48 (0.58~3.8)	0.74 (0.09~6.37)
	11–15 year	2.09 (0.8~5.49)	0.40 (0.04~4.08)	1.91 (0.74~4.96)	0.27 (0.03~2.76)
	16–20 year	2.02 (0.7~5.81)	–	1.99 (0.7~5.7)	–
	Over 20 years	1.0	1.0	1.0	1.0
Department	OED	1.34 (0.73~2.44)	2.25 (0.56~9.11)	1.39 (0.81 ~2.39)	2.73 (0.67~11.15)
	General surgery	2.31 (1.22~4.4)^a^	4.25 (0.93~19.33)	2.69 (1.48 ~4.89)^b^	2.71 (0.48~15.15)
	Intensive care	1.09 (0.49~2.45)	22.94 (2.9~181.23)^b^	1.16 (0.52 ~2.62)	30.21 (3.69~247.22)^b^
	Specialized unit	1.95 (1.01~3.76)^a^	9.34 (1.89~46.21)^b^	2.67 (1.50 ~4.78)^b^	15.68 (3.63~67.75)^b^
	Support services	0.18 (0.03~0.93)^a^	–	0.16 (0.03 ~0.86)^a^	–
	General medicine	1.0	1.0	1.0	1.0
Patients/ clients most frequently work with	Newborns	0.62 (0.19~2.08)	2.22 (0.44~11.15)		
	Non-newborns	1.0	1.0		
	Infants	7.21 (2.24~23.19)^b^	3.02 (0.58~15.7)		
	Non-infants	1.0	1.0		
	Children	2.52 (1.34~4.75)^b^	1.19 (0.32~4.47)		
	Non- children	1.0	1.0		
	Adolescents	1.44 (0.77~2.67)	2.06 (0.5~8.49)		
	Non-adolescents	1.0	1.0		
	Adults	1.69 (1.11~2.57)^a^	1.55 (0.53~4.54)		
	Non-adults	1.0	1.0		
	Elderly	1.66 (1.03~2.67)^a^	1.10 (0.3~4.06)		
	Non-elderly	1.0	1.0		
**Level-2**					
Measure (*n*)	Without measure (1)				3.05 (0.67~13.9)
	With measure (1)				1.0
	Without measure (13)			4.95 (2.16~11.37) ^b^	
	With measure (13)			1.0	
**Cross-Level Interactions**				
Measure (*n*) *Profession	Doctor*NO			1.31 (0.13~12.73)	11.26 (1.09~116.39)^a^
	Other HWs*NO			0.01 (0~0.45) ^a^	–
	Nurse*Yes			1.0	1.0

*Column heading shows dependent variable*.

*^a, b^denotes significance at the 5 percent and 1 percent level, respectively*.

*Measure (1), Security measures; Measure (13), Investment in human resource development*.

*PVV, patient and visitor violence*.

### Existing Measures Against Workplace Violence

The policies and measures against workplace violence implemented in the six hospitals in 2020, were reported by the respondents shown in Table 4. More than four-fifth (86.1%) of respondents reported that their workplace had implemented security measures, only 13.9% of them reported that their workplace had invested in human resource development, and 3.6% of them reported no measures at all. The prevalence of psychological and physical PVV for workplace where the measure against violence (MAV) existed and didn't exist are reported in Table 4, respectively. A combined multilevel regression model of Equation (I) and (II) was used to determine the association (the estimates of OR and 95% CI in Table 4) between each measure and PVV. The results indicate that after controlling for worker characteristics, the HWs in the workplace without measures of patient screening, increasing staff numbers, changing shifts or rotas, and investment in human resource development were found to be 2.6 (95% CI = 1.70~4.00), 1.8 (95% CI = 1.16~2.69), 1.8 (95% CI = 1.05~2.99) and 4.5 (95% CI = 2.14~9.38) times more likely to experience psychological PVV compared to HWs in the workplace with those measures, respectively. The HWs in the workplace without measures of security, improving surroundings, patient protocols, and investment in human resource development was found to be 7.9 (95% CI = 2.79~22.58), 4.4 (95% CI = 1.82~10.85), 4.7 (95% CI = 1.42~15.52), and 13.8 (95% CI = 1.32~143.21) times more likely to experience physical PVV compared to HWs in the workplace with the measures, respectively.

**Table 4:** The correlation between 13 measures against violence and PVV.

**Variables**	**Intervention measures**	**Respondents *n* (%)**	**Psychological PVV (%)**			**Physical PVV (%)**		
			**Yes**	**No**	***OR (95% CI)***	**Yes**	**No**	***OR (95% CI)***
Measure (1)	Security measures	649 (86.1)	30.4	19.0	0.65 (0.37~1.13)	2.9	11.4	7.93 (2.79~22.58)^b^
Measure (2)	Improve surroundings	536 (71.1)	30.8	23.9	0.8 (0.54~1.19)	2.6	7.8	4.44 (1.82~10.85)^b^
Measure (3)	Restrict public access	328 (43.5)	27.1	30.0	1.37 (0.95~1.97)	3.7	4.5	2.44 (0.99~6.02)
Measure (4)	Patient screening	224 (29.7)	17.9	33.4	2.61 (1.7~4.00)^b^	4.0	4.2	2.08 (0.81~5.32)
Measure (5)	Patient protocols	204 (27.1)	24.0	30.5	1.41 (0.93~2.12)	2.5	4.7	4.69 (1.42~15.52)^a^
Measure (6)	Restrict exchange of money at the workplace	188 (24.9)	24.5	30.2	1.36 (0.91~2.05)	2.7	4.6	2.05 (0.65~6.46)
Measure (7)	Increased staff numbers	225 (29.8)	20.9	32.1	1.77 (1.16~2.69)^b^	5.3	3.6	1.35 (0.52~3.52)
Measure (8)	Check-in procedures for staff	152 (20.2)	23.7	30.1	1.48 (0.94~2.33)	6.6	3.5	0.85 (0.34~2.14)
Measure (9)	Special equipment or clothing	151 (20.0)	23.8	30.0	1.52 (0.96~2.4)	4.0	4.1	1.35 (0.46~4)
Measure (10)	Changed shifts or rotas	127 (16.8)	18.9	30.8	1.77 (1.05~2.99)^a^	6.3	3.7	0.96 (0.34~2.71)
Measure (11)	Reduced periods of working alone	195 (25.9)	24.6	30.2	1.13 (0.75~1.7)	5.1	3.8	0.98 (0.39~2.49)
Measure (12)	Training	258 (34.2)	25.2	30.6	1.21 (0.84~1.75)	3.5	4.4	1.22 (0.49~3.06)
Measure (13)	Investment in human resource development	105 (13.9)	8.6	32.0	4.49 (2.14~9.38)^b^	1.0	4.6	13.77 (1.32~143.21)^a^

^a, b^*denotes significance at the 5 percent and 1 percent level, respectively*.

*OR (95% CI) were the results of multilevel logistic regression models*.

*PVV, patient and visitor violence*.

*Yes, The measure (n) existed in HWs' workplace; No, The measure (n) didn't exist in HWs' workplace*.

Model 2.1 and 2.2 in Table 3 shows the significant results of cross-level interactions between the measures and professions examined by a combined multilevel regression model of Equation (IV). There were significant interactions between the contextual variable *measure (1), measure (13)* and the individual level variable *profession*; in other words, the effect of *profession* does significantly vary for the workplace with and without the *measure (1), measure (13)*. Specifically, doctors in the workplace without security measures were 11.3 times (95% CI = 1.09~116.39) more likely to suffer from physical PVV compared to nurses in the workplace with security measures. And other HWs in the workplace without measures of investment in human resource development were less likely (OR = 0.01, 95% CI = 0.00~0.45) to suffer from psychological PVV compared to nurses in the workplace with those measures. No cross-level interactions were found between other measures and professions (Table 3).

## Discussion

This study is an investigation of PVV against HWs in multiple hospitals during the COVID-19 epidemic in China and is one of the few studies relating individual and contextual factors to PVV by multilevel regression analysis. The results of the study reveal that in the year of 2020, 29.2% of HWs experienced PVV in tertiary public hospitals from Beijing. Doctors were more likely to suffer from physical PVV than nurses. HWs most frequently work with infants were more likely to suffer from psychological PVV. More than four-fifth of respondents reported that their workplace had implemented security measures. The effect of *profession* does significantly vary between the workplace with and without the security measures, and the measure of investment in human resource development.

### The Situation of PVV in the Epidemic

This data set comes from a large representative sample of multiple hospitals. The findings confirm existing evidence that HWs in child hospitals were found to be about 1.5 times more likely to experience PVV compared to them in general hospitals (27). A recent published cross-sectional survey in China showed that the prevalence of WPV among mental health professionals in China during the COVID-19 pandemic (in 2 months) was 18.5% (16), which is higher than the occurrence rate of PVV (29.2% in a year) in this study from tertiary hospitals. However, the WPV from the former study not only includes the PVV, but also the horizontal violence in the workplace. Another study using the database of the Medical Quality and Safety Notification System showed that the overall prevalence of PVV for the 39 tertiary public hospitals in Beijing in 2015 was 16.6% (27), which is lower than the prevalence of PVV (29.2%) in this study that includes six tertiary public hospitals. In addition, the findings confirm existing evidence that HWs in some specialized hospitals (such as child hospital) had higher risk of PVV compared with general hospitals, and more attention should be paid to the workplace violence in child hospitals.

How to understand the prevalence of PVV in this study? In 2020, the COVID-19 pandemic ravaged all over the world. The COVID-19 broke out in Beijing from 11 June to 19 July with hundreds of people infected, and had been effectively controlled within a month. However, as the capital of China and international exchange center, Beijing is under great pressure to prevent and control the epidemic. Since February 2020, Beijing has issued a series of prevention and control measures. In the health sectors, different levels of COVID-19-specific IPC measures were implemented in hospitals, based on the local epidemiological situation, the specificity of the work setting and work tasks (28). For example, introduce measures for avoiding crowding and social mixing; restricting visitors; requirement of health pass codes and a negative nucleic acid certificate etc. Although these measures effectively protect HWs, patients and visitors from infection, the change of medical treatment process and visits regulation could also influence patients' intention to seek treatment from hospitals, and increase the risk of clinician-patient conflicts to some extent. According to the data of the Health Statistical Yearbook from Beijing municipal health commission, the total number of outpatient visits of tertiary hospitals in 2020 was 32% lower than that in 2019 (20, 29). The study of Huang and Zhang (27) shows that the prevalence of PVV was significantly positively correlated with the outpatient workload of doctors (β = 0.24, *p* < 0.01) in China. This means the PVV should have decreased during the 2020 due to the decrease in workload of doctors. However, long patient waiting times, IPC measures, and contact tracing etc. may also increase the risk of PVV against HWs. Therefore, although the prevalence of PVV in this survey is lower than that in some studies, it cannot be simply concluded that the situation of PVV in Beijing in 2020 is more or less severe than that in other countries or before.

### Worker Characteristics and PVV

The findings confirm existing evidence that HWs most frequently working with infants and children had a higher risk of psychological violence (15, 30–32). Patients and their relatives in pediatrics are more anxious and sensitive than other departments, thus increasing the possibility of conflicts and workplace violence. Previous studies found that HWs in pediatrics were at an increasing risk of PVV, because of the long labor shortage of pediatricians in China and the more vulnerable and sensitive patients there (27). In this study, the proportion of nurses who frequently worked with children (12.7%) was higher than other HWs, which means that nurses usually have a higher risk of psychological PVV.

Interestingly, doctors are more likely to suffer physical PVV than nurses (21), despite the latter having close contact with the patients more frequently, which is consistent with other studies from China (33). Indeed, doctors are often victims of physical PVV, especially the terrible violence in China. Why are doctors, not nurses? Previous studies suggested that the root cause of doctor-patient conflicts is the issue of trust between doctors and patients in China (34). The insufficient level of doctor-patient trust leads patients to blame the deterioration of their health directly on doctors rather than nurses (34, 35). During the outbreak of COVID-19, more and more patients went to hospital when they were in severe or critically ill condition, due to the risk of infection in epidemic. It's important to note that the severe patients and their relatives will become more anxious and sensitive than moderate patients, thus increasing the possibility of conflicts between doctors and patients, and the risk of physical PVV.

### The Comprehensive Measures Against PVV

The workplace with measures of patient screening, increasing staff numbers, changing shifts or rotas, and investment in human resource development have a lower risk of psychological PVV. Meanwhile, the workplace with measures of security, improving surroundings, patient protocols, and investment in human resource development have a lower risk of physical PVV. It suggested that the measures in these hospitals could protect HWs from PVV to a certain extent. In 2020, a series of policies were timely issued and implemented by Beijing municipal government to protect the occupational health and safety of HWs, including the most comprehensive and rigorous prevention and control strategy against the epidemic, proactive measures of occupational health, and precaution strategies against occupational hazards (5, 6, 16, 36). Therefore, strict IPC measures and precaution strategies against WPV have been both strictly implemented. Our study showed that most of HWs reported the measures against workplace violence existed in their workplace, and 86.1% of them were security measures. What's more, the cross-level interactions between *profession* and *measure (1)* shows that without the security measures, doctors were 11.3 times more likely to suffer from physical PVV than nurses. Indeed, Beijing Municipal Public Security Bureau and Beijing Municipal Health Commission jointly issued the policies, *Regulations of Beijing Municipality on the administration of hospital safety*, and *List of prohibited and restricted items in hospitals of Beijing* in the year of 2020 (37). After that, 90% of the secondary and tertiary public hospitals in Beijing had set up a security check system, and more than 80% had installed devices which HWs can press the button to report to police, and 86 hospitals were equipped with face recognition system for patient screening (to record and be aware of previous aggressive behavior). And then, the number of hospital-related crimes was reduced by 10.8%, by August 2021 (8). According to the evidence from Italy, Ferorelli D et al. also confirm that comprehensive measures can reduce aggression to the detriment of HWs such as reporting events (38) which could activate the most suitable measures to prevent attacks (39). These demonstrated that the existing measures against WPV are still very important and effective to fight against the PVV during the pandemic, and comprehensive measures (measures of IPC and against WPV) should be implemented to mitigate hazards and protect the health, safety, and well-being of health workers.

## Limitation and Strength

Firstly, due to the cross-sectional design, the causal associations between variables are still unknown. Second, recall bias cannot be excluded, especially in psychological PVV which is more likely to be under reported. Third, HWs participated voluntarily during the COVID-19 epidemic, which could lead to selection bias. It is possible that HWs who experienced the PVV or had sufficient time were more likely to participate in the survey. And for population studies in COVID-19 epidemic, it is difficult to interview all HWs by face-to-face investigation. Nevertheless, the total subjects recruited from six hospitals are typical for HWs in the health sectors of Beijing. What's more, the international definitions and questionnaire on workplace violence used in this survey enhances the validation of international comparison, and the multilevel logistic model provide an appropriate analytical framework to explore the nature and extent of relationships at both micro and macro level, which could provide evidence for further studies on the intervention with comprehensive measures. To our knowledge, the study is one of a limited number of studies in China to verify the risk factors of PVV using the international technical tool based on multilevel regression analysis.

## Conclusion

During COVID-19 pandemic, a series of policies were issued and implemented for HWs in China to protect the occupational health and safety of HWs, not only implementing strict IPC measures, but also the measures against violence. The security measures are very important and effective to fight against the physical PVV during the pandemic with widespread risk factors for workplace violence. Comprehensive measures (measures of IPC and against WPV) should be implemented to mitigate hazards and protect the health, safety and well-being of health workers.

## Data Availability Statement

The raw data supporting the conclusions of this article will be made available by the authors, without undue reservation.

## Ethics Statement

This study was approved by Ethical Review Committee of Chinese Academy of Medical Sciences (IMICAMS/8/22/HREC). Written informed consent was obtained from all participants before the survey. To ensure anonymity, no names, or other identifiers were used.

## Author Contributions

JH conceived and designed the study, performed the data analysis, and modified the manuscript. Y-qG conducted the literature search, interpreted the data, and wrote the first draft. N-nX and X-jM conducted surveillance, collected data, and had full access to all of the data in the study. All authors made important contributions to subsequent drafts and have seen and approved the final version.

## Funding

This research was funded by the National Natural Science Foundation of China (Project Identification Code: 71804192). The funder had no influence on the study design, on the collection, analysis, and interpretation of data, on the writing of the report, or on the decision to submit the article for publication.

## Conflict of Interest

The authors declare that the research was conducted in the absence of any commercial or financial relationships that could be construed as a potential conflict of interest.

## Publisher's Note

All claims expressed in this article are solely those of the authors and do not necessarily represent those of their affiliated organizations, or those of the publisher, the editors and the reviewers. Any product that may be evaluated in this article, or claim that may be made by its manufacturer, is not guaranteed or endorsed by the publisher.

## Footnotes

^1^Level-2 equation of empty model follows: β_0*j*_ = γ_00_+μ_0*j*_.
